# Palliative in Painful Urination

**Published:** 1873-12

**Authors:** 


A PALLIATIVE IN PAINFUL URINATION.—In retention of urine from stricture, in hypertrophy of the prostate, after operations on the urethra, etc., Dr. Cazenavi recommends the employment of ice suppositories. A piece of ice, of oval shape and the size of a chestnut, is inserted in the rectum. The relief is prompt and most satisfactory to both patient and physician.

